# First lung transplant in Wuhan for a critical and elderly COVID‐19 patient

**DOI:** 10.1002/iid3.501

**Published:** 2021-09-01

**Authors:** Bo Wang, Jie Huang, Micheal Hsin, Jingyu Chen, Huiqing Lin

**Affiliations:** ^1^ Department of Thoracic Surgery Renmin Hospital of Wuhan University Wuhan Hubei Province China; ^2^ Department of Cardiothoracic Surgery Queen Mary Hospital Hong Kong China; ^3^ Wuxi Lung Transplant Center Wuxi People's Hospital Affiliated to Nanjing Medical University Wuxi Jiangsu China

**Keywords:** COVID‐19, lung transplant, pulmonary failure

## Abstract

**Introduction:**

We report the case of a 65‐year‐old Coronavirus disease 2019 (COVID‐19) patient with pneumonia and subsequent end‐stage pulmonary failure who required 63 days of mechanical ventilation (MV) and 62 days of extracorporeal membrane oxygenation (ECMO).

**Methods:**

On Day 45, a comprehensive interdisciplinary discussion on the best course of treatment resulted in the general consensus that his lungs would not recover. As such, he was evaluated and listed for a lung transplant.

**Results:**

We performed a bilateral lung transplant, and the patient was weaned off ECMO and MV postoperatively. This is the first report of lung transplants in patients with COVID‐19 in Wuhan.

**Conclusions:**

We suggest that a lung transplantation may be a viable treatment for patients with end‐stage pulmonary failure secondary to COVID‐19 in selected situations.

## Graphical abstract

Lung transplantation provided an opportunity for this very severe selected patient a chance to survival, which is meaningful

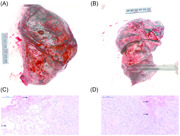

## 1 INTRODUCTION

On December 31, 2019, the World Health Organization (WHO) China Country Office was informed about cases of pneumonia of unknown etiology detected in Wuhan, the capital city of the Hubei province in China. Wuhan became the center of a pneumonia outbreak of unknown origin.^1^ In 2020, a pandemic novel coronavirus (2019‐nCoV) outbreak was declared by the WHO. During the first 2 months of the outbreak, 67 (6.1%) of 1099 patients with laboratory‐confirmed Coronavirus disease 2019 (COVID‐19) from 552 hospitals in 30 provinces in China were critically ill. Of this, 5.0% were admitted to the intensive care unit (ICU), 2.3% underwent invasive mechanical ventilation (MV), and 1.4% died.^2^ The complications of COVID‐19 are primarily manifested in the respiratory system culminating with end‐stage pulmonary failure and patients with end‐stage COVID‐19 have a higher risk of complications.^3^ Lung transplantation (LT) is an established treatment for end‐stage pulmonary failure. However, little is known about LT in cases of virus‐related diseases such as COVID‐19. Furthermore, bilateral LT in patients with end‐stage COVID‐19 disease has been reported in only a few anecdotal case. Herein, we report the case of a successful bilateral LT in a COVID‐19 patient with end‐stage pulmonary failure. The patient was on life support, namely, MV for 63 days and extracorporeal membrane oxygenation (ECMO) for 62 days before transplantation. We hope this report provides useful information regarding the management of elderly patients with COVID‐19 who are deemed to be in a critical condition.

## 2 CASE REPORT

A 65‐year‐old male who presented with a high temperature for 9 days and was admitted to his local hospital on February 1, 2020. A nasal swab specimen tested positive for severe acute respiratory syndrome coronavirus 2 (SARS‐CoV‐2) nucleic acid, and chest computed tomography (CT) revealed bilateral consolidation and ground‐glass opacities. His symptoms and radiographic manifestations worsened despite antiviral treatment with oral oseltamivir, intravenous cefuroxime, and proprietary Chinese medicine. He was transferred to a COVID‐19 designated hospital on February 7 for further treatment. On February 16, he was admitted to the intensive care unit (ICU) for noninvasive respiratory support, which was escalated to MV on February 17. Venovenous (VV)‐ECMO support commenced on February 18. The patient was transferred to our hospital on March 18 for comprehensive treatment. From March 18 to April 9, his diagnoses included sepsis, coagulation disorders, gastrointestinal bleeding, pneumothorax, and hemothorax. Coagulation disorders were corrected with intravenous blood products and, somatostatin, and pleural complications were treated with tube thoracostomy chest. On March 20, the patient was diagnosed with septic shock due to carbapenem‐resistant Acinetobacter baumannii (CRAB). Following antiviral, anti‐infective, immunosupportive, and life support therapy, produced five consecutive negative results for nucleic acid detection by the reverse transcriptase polymerase chain reaction (RT‐PCR) test for SARS‐CoV‐2, and three results of negative Immunoglobulin (Ig)M and positive IgG antibodies since February 28. Despite these efforts, his pulmonary function continued to deteriorate. The lung volume was only 220 ml and the static compliance 13 ml/cmH_2_O. As such, we judged that the patient could not be weaned off VV‐ECMO and MV despite prolonged support. Following a discussion among professionals from multidisciplinary teams at the hospital, it was concluded that a LT should be considered. After we told the outcome of our discussion to the patient and his family, they requested aggressive treatment. The case was approved by the ethics committee of the Renmin Hospital of Wuhan University and the patient was registered with the China Organ Transplant Response System (COTRS).

**Figure 1:**
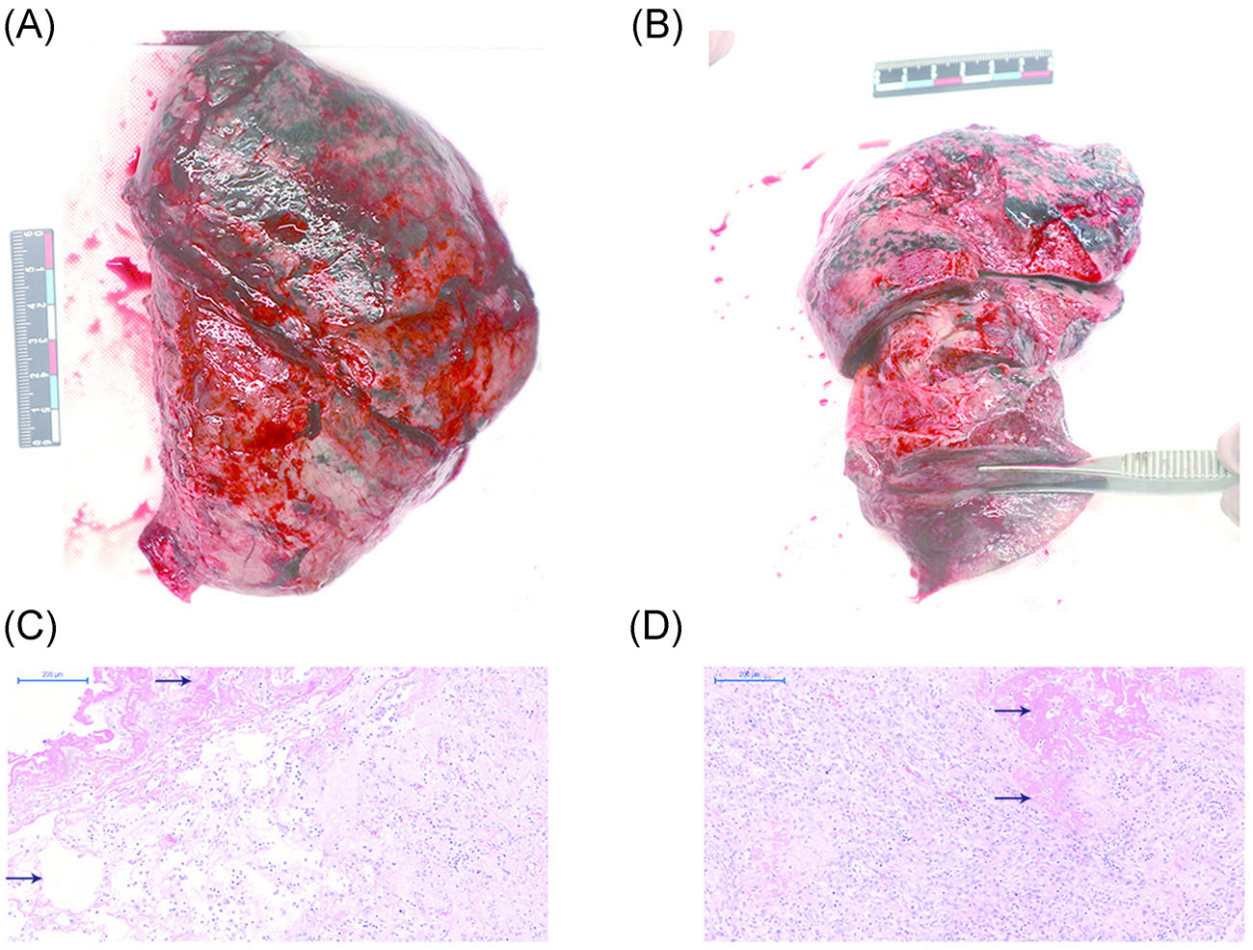
Gross and hematoxylin‐eosin staining photos of patients. Resected left (A) and right (B) lungs of the patient. (C, D) Pathological manifestations of lung tissue in the patient with severe pneumonia caused by SARS‐CoV‐2. Diffuse Alveoli collapses and fibrosis were showed in figures C and D (H&E ×100). The septa of the remained alveoli were slightly thickened with inflammatory cells and fibroblasts, and marked fibrin exudation (at the left upper margin) was observed in figure C. Fibrin exudatation could also be observed in the right upper field of figure D. H&E, hematoxylin‐eosin stain; SARS‐CoV‐2, severe acute respiratory syndrome coronavirus 2

After 2 weeks on the waiting list, a suitable donor was allocated by Chinese Donor Allocation System COTAS with the following characteristics: 32‐year‐old male; weight, 58 kg; height, 167 cm; matching blood type; clear chest radiograph; no history of smoking, malignancy; and PaO_2_/FiO_2_ ratio of 578 mmHg. Left LT was performed first with an ischemic time of 7.5 h followed by right LT with an ischemic time of 10 h. When suitable donor lungs became available, surgeons performed a clamshell thoracotomy for bilateral sequential LT. The incision connected the bilateral anterolateral thoracotomy incisions across the midline by dividing the sternum. The bilateral thoracic cavity contained approximately 1000 ml of yellow pleural effusion. Both mammary arteries are divided in this approach, and this incision provides added exposure during a concomitant cardiac procedure. A prior CT scan indicated that the right lung function was better than the left. Therefore, the left single LT was performed first via a standard procedure. After blocking the left main pulmonary artery before resecting the left lung, the heart rate and blood pressure decreased suddenly. The patient developed hemodynamic instability following occlusion of the left pulmonary artery, and central VA‐ECMO was established. We then instituted venoarterial (VA)‐ECMO (3 L/min) via an ascending aortic cannula (21F) and a single‐stage cannula in the right atrium, and downregulated the VV‐ECMO to 1.5 L/min. We administered 500 mg methylprednisolone before opening the pulmonary artery. The left donor lung expanded well without obvious edema. The surgeons proceeded to right pneumonectomy and implantation of the second donor lung. Right LT was performed via a standard procedure. On the left LT, perform the bronchial anastomosis with a 4‐0 polydioxanone running suture. Cover the anastomosis with local pericardial and parabronchial connective tissues. Next, anastomose the pulmonary artery using a running suture of 5‐0 polypropylene (Prolene). Take care to ensure that this graft is not too long or twisted. Retract the two tied pulmonary veins away from the heart, and place the Satinsky clamp at the base of the pulmonary veins. The postoperative blood pressure and heart rate were stable. The cold ischemia time for the left and right lung were 7.5 and 10 h, respectively. On gross inspection, the native lungs had alternating areas of black, gray, and dark red discoloration, and were firm on palpation (Figure 1A, B). The patient was weaned off VA‐ECMO support at the end of the procedure, and the VV‐ECMO support was retained while the patient was transferred to the ICU. The VV‐ECMO was successfully weaned off 44 h postoperatively. Propofol and sufentanil were administered postoperatively to maintain the Richmond Agitation‐Sedation Scale (RASS) score from −3 to −2 as the patient was cannulated with ECMO, an endotracheal tube, a central line, and a Foley catheter. After the patient was weaned off ECMO, sedation with propofol and sufentanil was gradually reduced and dexmedetomidine was used to maintain the RASS score from −1 to 0 to facilitate early rehabilitation. The patient was diagnosed with Acinetobacter baumannii bacteremia in the early postoperative stage. He then contracted *Pseudomonas aeruginosa* and subsequently Klebsiella isolated from bronchoalveolar lavage (BAL) fluid. With each infection, the causative pathogens were detected, by appropriate laboratory testing, bacterial susceptibility tests were performed promptly, and the anti‐infective treatment adjusted accordingly. We closely monitored procalcitonin (PCT), C‐reactive protein, body temperature, and other infection markers daily.

**Figure 2:**
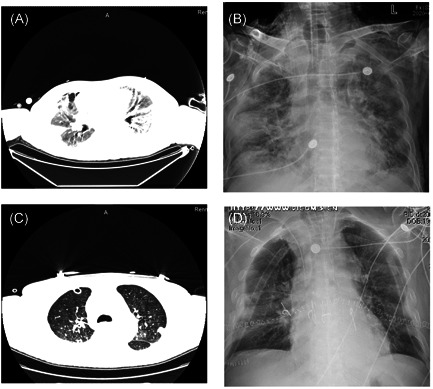
Chest CT and X‐ray image of patients. (A, B) The patient's chest CT before lung transplantation. (C, D) The patient's chest X‐ray on the fifth day after lung transplantation. CT, computed tomography

The patient was extubated on the 16th day after the LT. The pre‐ and posttransplant chest CT scans are shown in Figure 2. The patient was placed on a immunosuppression protocol of cyclosporin and prednisone. He started to receive a double regimen on the day before surgery (tacrolimus 0.1 mg/kg po bid), and prednisone (0.5 mg/kg po qd, tapering down by 5 mg/week to 0.25 mg/kg/day). The postoperative chest CT revealed clear lung fields with no infiltrates.

A rehabilitation program that included neuromuscular electrical stimulation, proprioceptive stimulation, joint activity training, limb movement, sitting, balance training, and muscle strength training was initiated to recover physical function. Breathing training was added to rehabilitation regimen following intermittent weaning of MV. Finally, the patient recovered to nearly Grade 4 muscle strength at discharge (Table 1). The patient died 4 months after discharge at last. As a result of prolonged supportive treatment, the patient had multiple organ impairments and eventually died of renal failure and its associated complications.

**Table 1:** Timeline from onset of illness, to transplant, to discharge from hospital

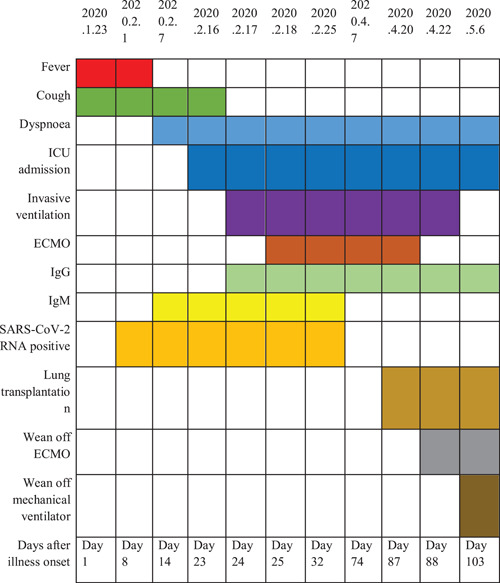

### 2.1 RT‐PCR test and histological results for SARS‐CoV‐2 in lung tissue

The explanted native lungs were sent to the Physical Containment Level 3 Laboratory. Pathological sections of the lungs from each lobe submitted for RT‐PCR for the detection of SARS‐CoV‐2 were negative. Histological examination indicated bilateral diffuse alveolar damage with fibrosis (Figure 1C, D). The septa of the remained alveoli were slightly thickened with inflammatory cells and fibroblasts, and marked fibrin exudation (at the left upper margin) was observed in Figure 1C. In Figure 1D, Fibrin exudatation could also be observed in the right upper field.

## 3 DISCUSSION

LT has been an established treatment for end‐stage pulmonary failure since the first successful case in 1983.^4^ However, cases of LT in virus‐related diseases are rare.^5^, ^6^, ^7^, ^8^, ^9^ In this report, we present the case of a successful bilateral LT for end‐stage pulmonary failure secondary to pneumonia as a result of COVID‐19 in a patient who required pretransplant support, with 63 days of MV and 62 of VV‐ECMO.

When the COVID‐19 pandemic occurred in Wuhan, the city entered a state of lockdown, and all organ donation and transplantation activities were postponed because of the high risk of potential infection. Transplants were expected to overwhelm the already understaffed public health system, especially in ICUs. This LT was performed when COVID‐19 was well controlled in Wuhan and considered as a low‐risk epidemic area. The IgM–IgG test is an accurate and sensitive diagnostic method. A combination of nucleic acid and IgM–IgG testing is a more sensitive and accurate approach for diagnosis and early treatment of COVID‐19. A positive IgM antibody indicates that the duration of infection is short, in this period we think is not suit for surgery. The coronavirus lockdown ended and Wuhan reopened on April 8, 2020, following which medical resources in Wuhan became sufficient to consider organ transplantation.

LT may be a viable alternative for patients with end‐stage pulmonary failure secondary to COVID‐19 in selective situations. We considered the following criteria as suitable for selecting LT recipients for end‐stage pulmonary failure secondary to COVID‐19 at our center^1^: no serious pre‐existing medical conditions;^2^ the course of clinical diagnosis and treatment of COVID‐19 infection exceeds 2 months, with proven recovery;^3^ the presence of irreversible pulmonary fibrosis and respiratory failure secondary to COVID‐19;^4^ dependence on ECMO and/or MV for at least 1 month, with no signs of improvement to aid weaning;^5^ three consecutive negative RT‐PCR nucleic acid tests for SARS‐CoV‐2 from a nasopharyngeal swab, an anal swab, sputum, BAL and blood;^6^ normal immunological function;^7^ normal neurological examination after discontinuation of sedatives;^8^ major organs (heart, liver, and kidneys) are functioning;^9^ normal coagulation function;^10^ no bacteremia or infection with drug‐resistant bacteria before surgery; and^11^ general condition deemed to have adequate rehabilitation potential following LT.

Notably, the real‐time RT‐PCR for SARS‐CoV‐2 was an important diagnostic indicator and was performed pretransplantation on samples from nasopharyngeal swabs and BAL. The WHO guidelines define laboratory confirmation of SARS‐CoV‐2 as a positive RT‐PCR of a nasal or pharyngeal swab.^10^ Cypel and Keshavjee suggest that the patient should have a recent negative SARS‐CoV‐2 PCR result, or infectivity assays using deep respiratory tract samples that show the absence of a viable virus before LT.^11^ Lang et al.^12^ reported the first case of a LT for a patient with a persistently positive SARS‐CoV‐2 real‐time RT‐PCR result. However, the viral culture was negative and the real‐time RT‐PCR *C*_t_ values were high. Chen et al.^13^ have reported the first case series on LT for COVID‐19 patients. They stipulated that at least three consecutive negative RT‐PCR nucleic acid tests for SARS‐CoV‐2 from a nasopharyngeal swab, an anal swab, sputum, BAL, and blood should be obtained owing to the potential of a negative RT‐PCR nucleic acid test to return to positive. The criteria proposed for patient selection and timing of LT in this study require validation in future studies. Furthermore, the current criteria should include confirmation of the absence of viable virus from nasopharyngeal and anal swabs, sputum, BAL specimens, and blood. A combination of nucleic acid and IgM–IgG testing is a more sensitive and accurate approach for diagnosis and early treatment of COVID‐19.

Our patient met the above criteria. The COVID‐19 infection was confirmed for over 80 days before assessing his suitability for a LT. His CT image revealed presumptive evidence of pulmonary fibrosis. The patient was supported by VV‐ECMO for 62 days and MV for 63 days and we were satisfied that he could not be weaned off. Three consecutive SARS‐CoV‐2 nucleic acid tests were negative. The patient had normal cellular and humoral immunity and a normal neurological examination. Bacteremia and other organ damage were absent (Table 2).

**Table 2:** Clinical and laboratory characteristics in the recipients before lung transplant and transfer while on ECMO

	Patient		
*Clinical characteristics*			
Age	65		
Gender	Male		
Epidemiological history	Yes (exposure to relevant environment)		
Date of admission	February 1		
EF（%）	60		
Signs and symptoms			
Fever on admission Cough Sore throat Diarrhoea Chest pain Myalgia Dyspnoea PRA‐I PRA‐II HLA‐A HLA‐B HLA‐DR	Yes Yes Yes Yes Yes No Yes 10.7% 5.5% A1A11 B37B55 DR12DR15		

Laboratory characteristics	Before LTx	Transfer while on ECMO	Range
ALT (U/L)	12	26	0–40
AST (U/L) PRO‐BNP (pg/ml) CK‐MB (ng/ml) Myoglobin (μg/L) TNI (ng/ml) Ur (mmol/L) Cre (μmmol/L) GFR (ml/min) PT (sec) INR Dimer (mg/L) CD3 (%) CD4 (%) CD8 (%) CD19 (%) CD16+56 (%) IgG (g/L) IgM (g/L) IgA (g/L) IgE (IU/ml) C3 (g/L) C4 (g/L)	15 1683 0.75 39.88 0.017 13.1 98 69.42 13.4 1.15 9.24 76.76 46.86 28.44 4.89 17.79 7.35 0.561 0.975 55.6 0.667 0.207	35 2000 0.93 43.52 0.023 28.5 75 92 15.2 1.32 1.35 65.9 34.94 31.01 2.53 31.31 6.88 0.638 0.728 19.2 0.552 0.198	0–40 0–450 0–5 0–110 0–0.04 3.6–9.5 57–111 >90 9–13 0.76–1.24 0–0.55 56–86 33–58 13–39 5–22 5–26 8.6–17.4 0.3–2.2 1.0–4.2 <100 0.7–1.4 0.1–0.4

Abbreviations: ALT, Alanine aminotransferase; ARDS, acuterespiratrydistresssyndrme; AST, Aspartate aminotransferase; CK‐MB, creatine phosphokinase‐MB; Cre, Creatinine; ECMO, extracorporeal membrane oxygenation; EF, ejection fraction; GFR, Glomerular filtration rate; HLA, Human leukocyte antigen; IgA, immunoglobulin A; INR, International normalized ratio; PRA, panel reactive antibodies; PRO‐BNP, pro brain natriuretic peptide; PT, Prothrombin time; TNI, Troponin I; Ur, Urea.

Recognizing the limitations of testing and the possibility of atypical symptoms, we applied an even higher clinical index of suspicion. Utilization of Grade 3 personal protective equipment (PPE), including an intraoperative positive pressure mask, presented a challenge for surgeons, anesthesiologists, and nurses, whose range of motion was limited. Additionally, the health workers were unable to communicate effectively during the operative process, which made the operation more difficult and required tacit understanding. Sign language and preoperative rehearsals were also utilized to ensure clear understanding by all team members of the different scenarios. Reassuringly, the RT‐PCR results for SARS‐CoV‐2 taken from different lung lobes were negative, demonstrating that there was no virus replication. Decreasing the level of PPE when operating on a known SARS‐CoV‐2 patient with negative nucleic acid tests warrants further investigation.

Patients with severe COVID‐19 often progress to acute respiratory failure, and ongoing inflammation often inflammation results in pulmonary damage and subsequent multiple organ failure.^14^ Protecting the function of multiple organs is essential during the course of treatment. Intraoperative ECMO and postoperative continuous renal replacement therapy (CRRT) were used to protect the cardiac and renal function. The cardiac function by echocardiography was normal preoperatively. However, it was considered that concomitant cardiovascular procedure might be required. ECMO is believed to contribute to lung function recovery in severely infected patients and serves as an interim treatment and bridge before LT to prevent mortality. LT in SARS patients with ARDS has been reported in anecdotal case reports. However, LT has been performed for patients receiving ECMO for >45 days after H1N1 infection.^15^ Several reports suggest that LT should not be considered before 4–6 weeks of ECMO support after the initial clinical signs of respiratory failure. The VA‐ECMO was utilized during LT to reduce cardiac load and to facilitate gas exchange. ECMO was chosen instead of cardiopulmonary bypass for intraoperative support because^1^ ECMO results in less damage to red blood cells;^2^ No additional anticoagulation is required during ECMO; and^3^ so that we can continue to use ECMO to support cardiac dysfunction and hypoxemia when the patient returns to the ICU after LT. Meticulous attention to enteral nutrition, physical therapy, deep vein thrombosis prophylaxis, and daily evaluation of organ functions is essential for patients requiring prolonged ECMO support.^8^ VV‐ECMO was weaned off 44 h after the LT in our case. CRRT was used for nonrenal indications during the LT, and helped to remove inflammatory mediators such as cytokines and maintain a negative fluid balance,^16^ thereby reducing the occurrence of primary graft dysfunction (PGD). The 62 days of preoperative ECMO and the influence of COVID‐19 impaired the patient's coagulation ability. As a result, he lost nearly 7000 ml of blood intraoperatively. We infused over 10,000 ml of fluid including crystalloids, colloids, and blood products. Postoperatively, we detected severe pulmonary edema and decreased lung compliance. To maintain the negative fluid balance we initiated CRRT, which is very effective for improving pulmonary edema and lung compliance. Following the procedure rehabilitation commenced to facilitate earlier recovery. This included physical exercise, neuromuscular electrical stimulation, and oral tactile stimulation.

Patients with viral infections are usually at risk of multiorgan damage, as well as immune system impairment.^17^ COVID‐19 can attack the immune system, creating a characteristically low lymphocyte count. As such, immunosuppressed patients are at increased risk of severe disease from COVID‐19.^18^ Furthermore, it can be difficult to determine an appropriate balance between immunosuppressive and anti‐infectious measures. Given that such COVID‐19 patients may be sensitive to immunosuppressive drugs, we selected our immunosuppressive protocol prudently and used them at a lower dosage. Cyclosporin A (CsA) is a well‐known immunosuppressive drug that binds to cellular cyclophilins to inhibit calcineurin^19^ and reportedly inhibits the replication of coronaviruses.^20^ CsA is easily adjusted according to immune status due to its wide therapeutic range; thus, we used CsA and prednisone for immunosuppression.

In conclusion, to our knowledge, we reported the first successful LT for an elderly and critical COVID‐19 patient with end‐stage pulmonary fibrosis secondary to pneumonia in Wuhan. This patient received ECMO support for 62 days indicating a high risk of mortality. ECMO was weaned off 44 h after LT and MV was weaned off after the 16th postoperative day. As LT is not routinely performed in pulmonary failure occurring secondary to pneumonia, we do not yet know the long‐term outcome in this group of COVID‐19 patients, however, our patient remains well at the time of writing. Despite the limited evidence of case reports from a similar situation, it is important to emphasize that LT provided an opportunity for recovery and survival in this patient.

## AUTHOR CONTRIBUTIONS

Bo Wang, Jie Huang contributed to conception, designed experiments and were responsible for the whole work; Micheal Hsin, Jingyu Chen, Huiqing Lin analyzed experimental results and wrote the manuscript. All authors contributed to data analysis, drafting or revising the article, have agreed on the journal to which the article will be submitted, gave final approval for the version to be published, and agree to be accountable for all aspects of the work.

## Data Availability

All data included in this study are available upon request by contact with the corresponding author.
